# Ultrasound-guided screw fixation for patellar stress fracture in a female basketball player: A case report

**DOI:** 10.1097/MD.0000000000030040

**Published:** 2022-08-19

**Authors:** Toru Omodani, Kenji Takahashi, Keishin Ueno

**Affiliations:** a Sports Medicine & Joint Center, Funabashi Orthopedic Hospital, Funabashi, Japan; b Department of Physical Medicine and Rehabilitation, University of Pittsburgh School of Medicine, Pittsburgh, PA.

**Keywords:** patellar stress fracture, ultrasonography, ultrasound, ultrasound-guided surgery

## Abstract

**Introduction:**

Stress fracture of the patella is an overuse disorder that occurs in the lower extremity. Surgery may be considered if conservative treatment does not work or if a quicker and more reliable return to sports is expected. Surgery is usually performed under fluoroscopic guidance based on the premise that the proper placement of the internal fixation material can be determined on radiographic images.

**Patient concerns:**

A 16-year-old female basketball player gradually became aware of pain in the anterior aspect of her right knee during basketball, without any particular history of trauma. A computed tomography scan revealed a small bone fragment on the lateral side of the distal end of the patella.

**Diagnosis:**

Stress fracture of the patella.

**Interventions:**

Since it was difficult to determine the exact location of the bone fragment on radiographs, the surgery was performed under ultrasound guidance rather than fluoroscopy. While checking the bone fragment with ultrasound images, insertion of the guide pin, drilling, and screw fixation were performed under ultrasound guidance.

**Outcomes:**

Postoperative computed tomography showed accurate screw insertion into the bone fragment. Two months after surgery, bony fusion was confirmed, and the patient returned to her preinjury level of basketball.

**Conclusion:**

Ultrasound-guided screw fixation was useful in this case in which internal fixation of fractures is difficult under fluoroscopic guidance.

## 1. Introduction

We present the case of a 16-year-old female basketball player who underwent ultrasound-guided screw fixation for a stress fracture of the patella. This case report presents a novel ultrasound-guided procedure for fractures.

## 2. Case report

A 16-year-old female basketball player with no particular history of trauma gradually became aware of pain in the anterior aspect of her right knee during basketball. Although she continued to play, the worsening pain made it difficult for her to do so at full strength. One month after the pain onset, the patient visited our hospital.

**Figure 1.**
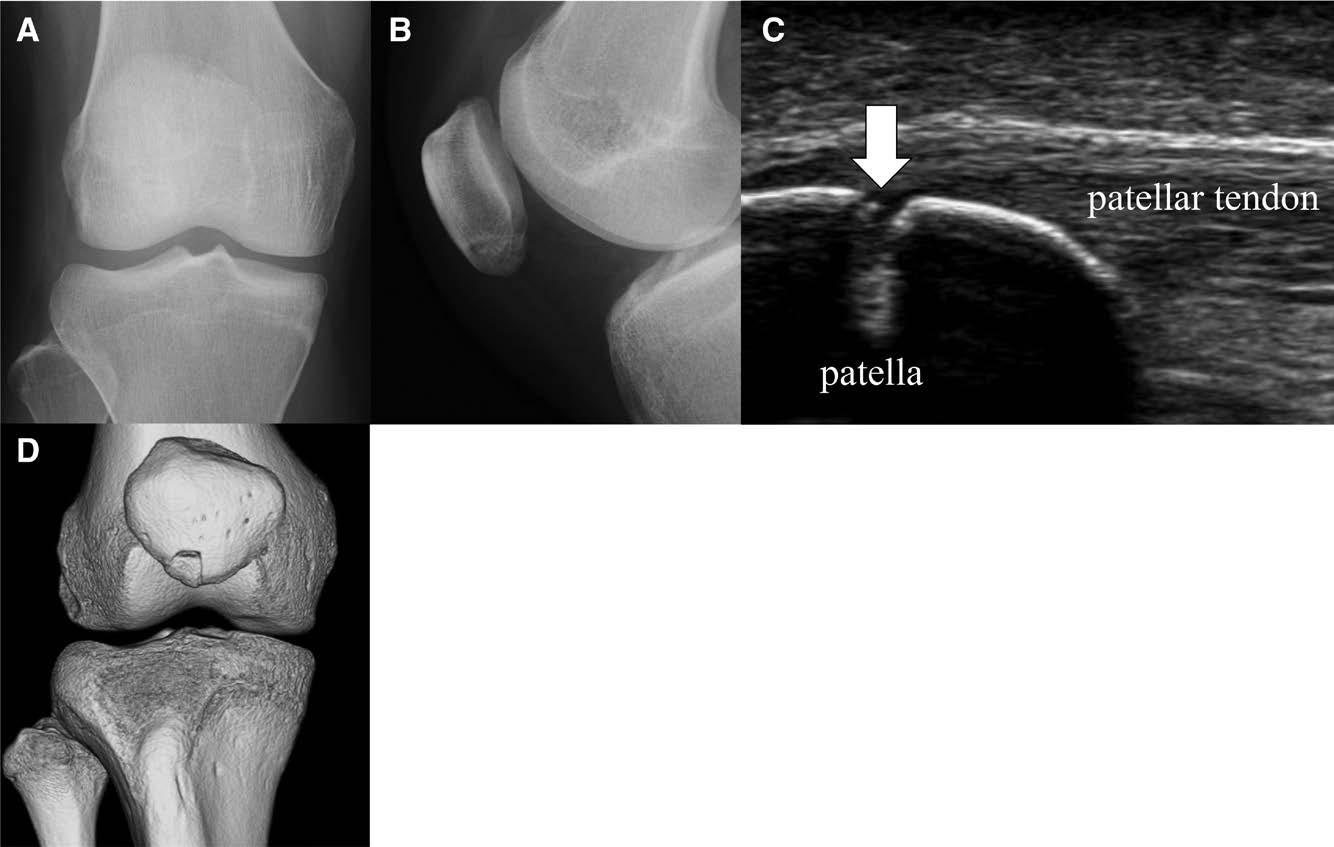
Imaging findings at the initial examination. (A, B) Radiographs. (C) Ultrasound. White arrow represents fracture. (D) Three-dimensional computed tomography.

Physical examination revealed tenderness at the distal end of the patella. Lateral plain radiographs showed linear translucent changes in the distal patella; however, frontal radiographs showed no obvious abnormalities (Fig. 1A, B). Ultrasonography showed interruption of the linear hyperechoic region, indicative of the patellar cortex, when the probe was placed in the longitudinal direction against the patella (Fig. 1C). Three-dimensional computed tomography showed a bone fragment on the lateral side of the distal end of the patella, which was dislocated from the main body of the patella (Fig. 1D). Each fragment measured 8 × 10 × 10 mm.

Based on these imaging findings, the patient was diagnosed with a stress fracture of the patella with dislocation of small bone fragments. The patient did not agree to stop playing basketball and rest and continued to play while undergoing rehabilitation at her own request. However, the pain worsened, and it became difficult for her to play basketball; therefore, surgery was performed 1 month after the initial consultation. As it was expected that it would be difficult to accurately grasp the position of the bone fragment and to insert the screw into the small fragment under fluoroscopy, we performed the surgery under ultrasound guidance.

**Figure 2.**
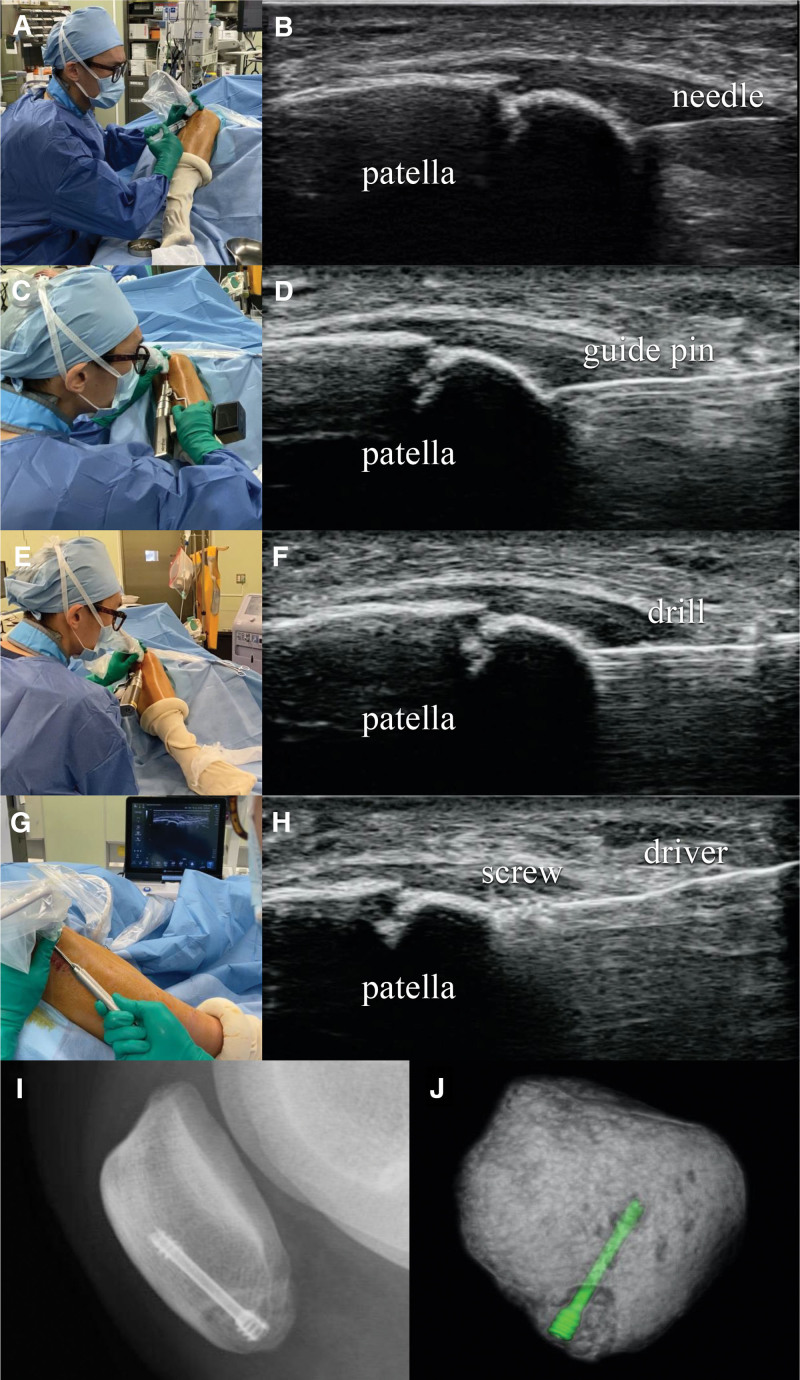
Surgical maneuvers and ultrasound images (A–H). (A, B) Simulated guide pin insertion site using a 25-gauge needle. (C, D) Guide pin insertion. (E, F) Drilling. (G, H) Screw insertion. (I) Radiographs taken immediately after surgery. (J) Three-dimensional computed tomography image taken 1 wk after surgery.

Surgery was performed under general anesthesia. The patient was placed in a supine position with the knee joint flexed at approximately 90° and a cushion placed behind the knee. An ultrasound probe was placed along the long axis of the patella to identify the fracture site. First, a 25-G needle was inserted from the distal to the proximal region under ultrasound guidance, and the position and direction of guide pin insertion were assumed (Fig. 2 A, B). After making a 4-mm skin incision with a scalpel, a guide pin 1.2 mm in diameter was inserted into the bone fragment and advanced to the contralateral cortex (Fig. 2C, D). A fluoroscope was used very briefly to confirm the direction of guide pin insertion. Next, drilling was performed with a 2.3-mm diameter drill (Fig. 2E, F). Finally, a double-thread headless screw (DTJ screw, Meira, Japan) with a proximal diameter of 4.0 mm, and length of 23 mm was inserted under ultrasound guidance until its tail was aligned with the surface of the bone fragment (Fig. 2G,H). The screw position was confirmed on radiographs, and surgery was completed by suturing the skin (Fig. 2I).

After surgery, no external fixation was performed, and range of motion training and loading of the knee joint were allowed according to the degree of pain. The stitches were removed 1 week after the surgery, and rehabilitation, including strength and balance training, was started. Computed tomography performed at the time of suture removal confirmed accurate screw insertion into the bone fragment (Fig. 2J). Three weeks after surgery, the fracture line tended to disappear on radiographs, and the patient resumed jogging. Two months postoperatively, the patient was able to run at full speed and join team practice. Bone fusion was confirmed by radiography. She participated in official games 4 months after surgery and continued to play basketball without any recurrence of the fracture, retiring from the club 10 months after surgery upon her graduation from high school. The patient reported slight knee pain after basketball.

## 3. Discussion

Stress fracture of the patella is an overuse disorder that occurs in the lower extremity and is believed to be caused by traction forces from the quadriceps and patellar tendons on the patella.^[1]^ It is an uncommon condition, with only 24 case reports so far.^[2]^ Surgery may be considered if conservative treatment does not work or if a quicker and more reliable return to sports is expected.^[3]^ Surgery is usually performed under fluoroscopic guidance based on the premise that the proper placement of the internal fixation material can be determined on radiographic images.^[1,4,5]^ The present case had a relatively small bone fragment confined to the lateral side of the distal end of the patella, making it difficult to accurately evaluate the lesion on radiographs; however, ultrasonography was able to accurately determine the location of the fragment.

In this case, the insertion of the guide pin, drilling, and screw fixation were performed under ultrasound guidance. The advantage of ultrasound-guided surgery is that it is minimally invasive because it does not require the development of the surgical field.^[6]^ Ultrasound-guided surgery may also allow for anatomically accurate procedures. Hattori et al^[7]^ performed an ultrasound-guided repair of the anterior talofibular ligament and revealed the anatomical accuracy of the anchor position to the fibula. In the present case, the surgical field did not need to be expanded to confirm the position of the bone fragment, and the operation was completed using only a small skin incision for screw insertion. In addition, the screw was inserted at the exact position of the small bone fragment.

Shen et al^[8]^ used ultrasound to confirm the alignment and position of the K-wire insertion during internal fixation of metacarpal fractures. They reported the radiation exposure time was reduced significantly. In the present case, fluoroscopy was used for only a very short time to confirm the direction of guide pin insertion under ultrasound guidance, and it was assumed that the radiation exposure was low.

Ultrasound-guided screw fixation for fractures has not been previously reported. In this case, surgery under ultrasound guidance allowed precise drilling and screw insertion while visualizing the fracture site. Ultrasound-guided surgery may be useful in cases in which internal fixation of fractures is difficult under fluoroscopic guidance.

## Author contributions

**Conceptualization:** Toru Omodani.

**Data curation:** Toru Omodani.

**Formal analysis:** Toru Omodani.

**Supervision:** Kenji Takahashi. Keishin Ueno.

**Writing:** Toru Omodani.
